# Editorial: Porous metal-organic framework (MOF) materials: design strategy, synthesis, sensing and catalysis

**DOI:** 10.3389/fchem.2023.1245159

**Published:** 2023-07-07

**Authors:** Lu-Fang Ma, Dong-Sheng Li, Guo-Ping Yang, Qichun Zhang

**Affiliations:** ^1^ Henan Key Laboratory of Function-Oriented Porous Materials, College of Chemistry and Chemical Engineering, Luoyang Normal University, Luoyang, China; ^2^ Key Laboratory of Inorganic Nonmetallic Crystalline and Energy Conversion Materials, College of Materials and Chemical Engineering, Hubei Provincial Collaborative Innovation Center for New Energy Microgrid, China Three Gorges University, Yichang, China; ^3^ Shaanxi Key Laboratory of Physico-Inorganic Chemistry, College of Chemistry and Materials, Science, Northwest University, Xi’an, China; ^4^ Department of Materials Science and Engineering, City University of Hong Kong, Kowloon, Hong Kong SAR, China

**Keywords:** metal-organic frameworks (MOFs), luminescence, defected MOFs, Cu(II) Ion doping, adsorption, fluorescence sensing

Many different kinds of organic (antibiotics, explosives, and dye, etc.) and inorganic (cationic and anion) pollutants in water can enter the human body through the food chain, which will lead to irreversible damage to human health (Srivastava et al., 2004; Joarder et al., 2015; Li et al., 2020). The rapid and sensitive identification or capture of organic/inorganic pollutants is therefore of great significance for protecting ecosystems and human health (Fu et al., 2018; Li et al., 2021; Tang et al., 2023). Porous metal-organic frameworks (MOFs) are synthesized by metal ions/clusters and various organic ligands via coordination bonds (Islamoglu et al., 2017). They have been proven to hold outstanding properties, such as ultra-high specific surface area, high porosity, and adjustable porous structures. Thus, MOFs may show a lot of potential for adsorbing and sensing environmental pollutants and are thought to be the most promising sorption and sensing materials (Rowsell and Yaghi, 2006; Mallick et al., 2015; Xing et al., 2021).

This Research Topic on “*Porous metal-organic framework (MOF) materials: design strategy, synthesis, sensing and catalysis*” includes recent studies on the characteristics and different applications of porous functional MOFs. The Research Topic consists of four original research papers from eight different institutions. Using the metallic cobalt ions and π-conjugated amide-functionalized ligands, Yan et al. prepared two nano-MOFs TMU-50 and TMU-51, both of which displayed dual interpenetrated frameworks and distinct luminescent properties. The authors studied the effects of some important parameters on the morphology and size of the nanostructures during the synthesis process, including the initial reagent concentration, ultrasonic power, and time. The BET results showed that TMU-51 is a non-porous motif, whereas the TMU-50 is a porous structure. Based on the unique porosity of TMU-50, it could be used for sensitively detecting nitroaromatics (NP) with a lower detection limit of 2 × 10^−5^ M, which had the equivalent ability to the reported luminescent MOF–based sensors. The enhanced selectivity of nano-TMU-50 for NP is attributed to the electrostatic interactions between the functional amide group of the ligand and the hydroxyl unit of NP. Jia et al. reported a new complex Zn-MOF with good photophysical properties. The water stability and detection sensitivity of Zn-MOF could be greatly improved by doping Cu^2+^ ions via the one-pot strategy. The doped Cu_0.1_/Zn-MOF was explored as a fluorescent sensor and its detection performance was carefully investigated for various metal ions and antibiotics. The results indicated that the Cu_0.1_/Zn-MOF showed high sensitivity, low detection limit, good cycling stability, and fast response for the detection of Fe^3+^, nitrofurans, and tetracycline in the aqueous media. This study may provide some guidance for the design and preparation of some novel luminescent MOF materials. Hu et al. obtained a rare three-dimensional (3D) heterometallic hafnium-based *flu* topological MOF (NS-1) with two different metal clusters [Hf_6_(μ_3_-OH)_8_(OH)_8_]^8+^ and [Cu_4_I_4_]. Interestingly, the 3D network features a rhombus channel of 13.10 × 19.27 Å^2^, which may provide a potential possibility for the iodine molecules with a diameter of 3.35 Å to enter the voids. The research showed that the NS-1 exhibited excellent reversible sorption ability for the iodine in the cyclohexane solution. Additionally, it was found that the sorption behavior fitted well with the pseudo-second-order kinetics and the Freundlich model based on the multilayer sorption. Six new heterometallic AE-Ln coordination polymers (CPs) with 2D layered motifs have been successfully synthesized by Hou et al. It was noted that the AE-Eu-CPs had stronger fluorescence durations and quantum yields than those of the AE-Tb-CPs. This might be explained by the fact that the energy match of 2,3-naphthalenedicarboxylic acid with Eu^3+^ could sensitize the luminescence of the Eu^3+^ ion but not with Tb^3+^, which resulted in the weak fluorescence of AE-Tb-CPs. The creation of (BaO)_n_ chains was found to have a more significant impact on the fluorescence amplification when the effects of three distinct alkaline Earth metal ions on the fluorescence were evaluated. Additionally, these complexes could be used as fluorescent probes for Fe^3+^ ions in aqueous solutions. The fluorescence quenching mechanism may cause weak interactions between complexes and Fe^3+^ ions, which hinders the passage of energy from the ligands to the Ln^3+^ ions and results in emission quenching finally.

We are thrilled to present the four peer-reviewed articles in this Research Topic to researchers in the field. These articles may highlight the non-traditional synthesis methods of functional MOF materials and the construction and applications of heterometallic MOF materials. We sincerely hope that readers gain valuable research information from these articles and that it instigates new ideas for ongoing progress in the field of MOF materials in the future.

## References

[B1] Fu H. R. Yan L. B. Wu N. T. Ma L. F. Zang S. Q. (2018). Dual-emission MOF⊃dye sensor for ratiometric fluorescence recognition of RDX and detection of a broad class of nitro-compounds. J. Mat. Chem. A 6, 9183–9191. 10.1039/c8ta02857e

[B2] Islamoglu T. Goswami S. Li Z. Howarth A. J. Farha O. K. Hupp J. T. (2017). Postsynthetic tuning of metal–organic frameworks for targeted applications. Acc. Chem. Res. 50 (4), 805–813. 10.1021/acs.accounts.6b00577 28177217

[B3] Joarder B. Desai A. V. Samanta P. Mukherjee S. Ghosh S. K. (2015). Selective and sensitive aqueous-phase detection of 2,4,6-Trinitrophenol (TNP) by an amine-functionalized metal–organic framework. Chem. Eur. J. 21, 965–969. 10.1002/chem.201405167 25424400

[B4] Li C. Yang W. Zhang X. Han Y. Tang W. Yue T. (2020). A 3D hierarchical dual-metal–organic framework heterostructure up-regulating the pre-concentration effect for ultrasensitive fluorescence detection of tetracycline antibiotics. J. Mat. Chem. C 8, 2054–2064. 10.1039/c9tc05941e

[B5] Li Y. An J. D. Wang T. T. Shi Y. F. Huo J. Z. Wu X. X. (2021). An ultra-stable cadmium(II) coordination framework constructed from the new bi-functional ligand and application as fluorescent probe for acetylacetone and antibiotics. Dyes Pigments 186, 109039. 10.1016/j.dyepig.2020.109039

[B6] Mallick A. Garai B. Addicoat M. A. Petkov P. S. Heine T. Banerjee R. (2015). Solid state organic amine detection in a photochromic porous metal organic framework. Chem. Sci. 6, 1420–1425. 10.1039/c4sc03224a 29560230 PMC5811159

[B7] Rowsell J. L. C. Yaghi O. M. (2006). Effects of functionalization, catenation, and variation of the metal oxide and organic linking units on the low-pressure hydrogen adsorption properties of metal−organic frameworks. J. Am. Chem. Soc. 128, 1304–1315. 10.1021/ja056639q 16433549

[B8] Srivastava S. Sinha R. Roy D. (2004). Toxicological effects of malachite green. Aquat. Toxicol. 66, 319–329. 10.1016/j.aquatox.2003.09.008 15129773

[B9] Tang Y. Lu X. M. Yang G. Wang Y. Y. (2023). Paddle-wheel-shaped porous Cu(II)−organic framework with two different channels as an absorbent for methylene blue. Inorg. Chem. 62, 1735–1743. 10.1021/acs.inorgchem.2c04350 36656916

[B10] Xing S. Liang J. Brandt P. Schäfer F. Nuhnen A. Heinen T. (2021). Capture and separation of SO_2_ traces in metal–organic frameworks via pre-synthetic pore environment tailoring by methyl groups. Angew. Chem. Int. Ed. 60, 17998–18005. 10.1002/anie.202105229 PMC8457122 34129750

